# Emphysematous Pancreatitis as a Life-Threatening Condition: A Case Report and Review of the Literature

**DOI:** 10.3390/medicina60030406

**Published:** 2024-02-27

**Authors:** Aleksandar Filipović, Dragan Mašulović, Dušan Bulatović, Miloš Zakošek, Aleksa Igić, Tamara Filipović

**Affiliations:** 1 Faculty of Medicine, University of Belgrade, 11000 Belgrade, Serbia; aleksandar.filipovic11@gmail.com (A.F.); draganmasulovic@yahoo.com (D.M.); zaki0502@gmail.com (M.Z.); 2 Center for Radiology, University Clinical Centre of Serbia, 11000 Belgrade, Serbia; dusan.bulatovic1@gmail.com (D.B.); aleksaigic95@gmail.com (A.I.); 3 Institute for Rehabilitation, 11000 Belgrade, Serbia

**Keywords:** emphysematous, acute pancreatitis, necrotizing pancreatitis, infection, computed tomography, surgery

## Abstract

Emphysematous pancreatitis represents the presence of gas within or around the pancreas on the ground of necrotizing pancreatitis due to superinfection with gas-forming bacteria. This entity is diagnosed on clinical grounds and on the basis of radiologic findings. Computed tomography is the preferred imaging modality used to detect this life-threating condition. The management of emphysematous pancreatitis consists of conservative measures, image-guided percutaneous catheter drainage or endoscopic therapy, and surgical intervention, which is delayed as long as possible and undertaken only in patients who continue to deteriorate despite conservative management. Due to its high mortality rate, early and prompt recognition and treatment of emphysematous pancreatitis are crucial and require individualized treatment with the involvement of a multidisciplinary team. Here, we present a case of emphysematous pancreatitis as an unusual occurrence and discuss disease features and treatment options in order to facilitate diagnostics and therapy.

## 1. Introduction

Acute pancreatitis is a commonly seen emergency in daily clinical practice with a wide range of severity degrees. Emphysematous pancreatitis (EP) is an uncommon variant of acute necrotizing pancreatitis characterized by the gas within or around pancreatic necrosis [1]. Prognosis is extremely poor, and early radiological detection may influence survival [2]. Diagnosis is established typically by computed tomography (CT) scanning in the appropriate clinical setting, which reveals characteristic findings of parenchymal nonenhancement with intrapancreatic or peripancreatic gas and fluid collections [3]. CT scan shows gas inclusions within the pancreatic bed, as well as the degree of pancreatic inflammation and possible complications of this severe form of acute necrotizing pancreatitis [2]. The management of EP includes conservative therapy, such as administering fluids, electrolytes, and antimicrobial therapy to control septic shock. Depending on the response to conservative measures and clinical condition of the patient, surgical debridement or percutaneous drainage may also be feasible [2].

The aim of this report is to present a case of emphysematous pancreatitis as an unusual occurrence, as the early diagnosis of EP is critical, and clinicians should consider this potentially fatal subtype of severe acute necrotizing pancreatitis when assessing patients with symptoms of acute pancreatitis and gas in the pancreatic bed. The overall prognosis of these patients remains poor, emphasizing the challenges in the management of these cases. We discuss disease features and treatment options in order to facilitate diagnostics, therapy, and to contribute our experience to the pool of data.

## 2. Case Presentation

**Figure 1:**
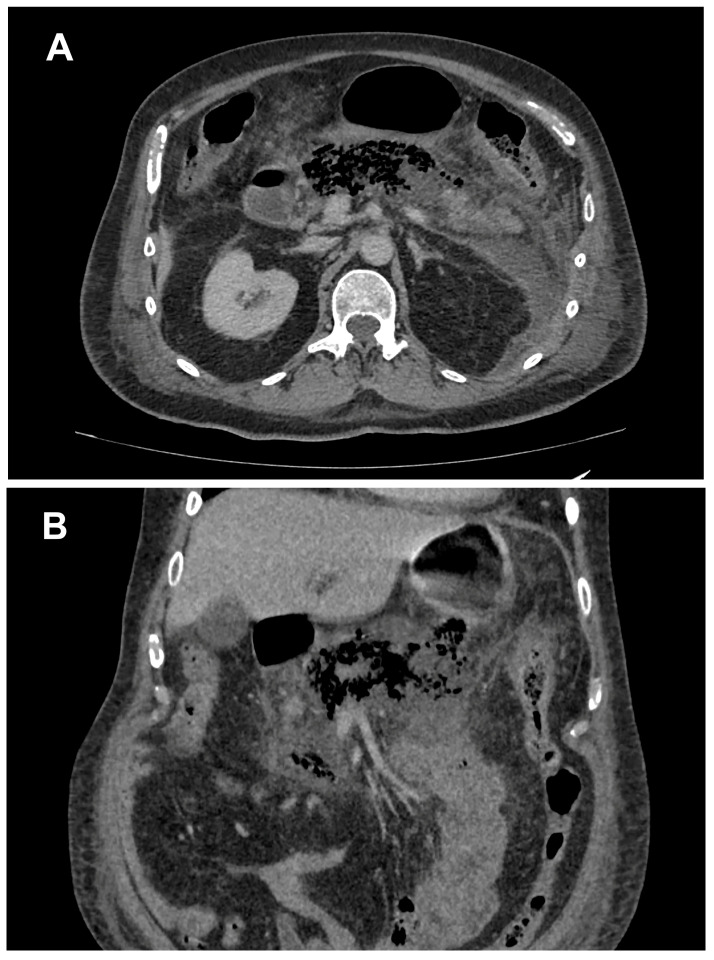
Contrast-enhanced computed tomography, axial (**A**) and coronal (**B**) reconstruction, venous phase of the exam showing gas in the body and tail of the pancreas. Pancreatic exudate in the left anterior pararenal space with surrounding retroperitoneal fat stranding is also noted.

A 64-year-old man was admitted to our emergency department due to progressively worsening abdominal pain with propagation into the chest. The pain was initiated in the epigastrium and accompanied with vomiting and diarrhea of several days’ duration. His past medical history included type I diabetes, hyperlipidemia, cholecystitis, gastritis, hypertension, and cardiomyopathy. He did not consume alcohol. On examination, the patient presented with hypotension (blood pressure 110/50 mmHg), atrial fibrillation (heart rate 150/min), ECG abnormalities such as ST elevation, and he had a distended abdomen with mild epigastric tenderness. Laboratory tests showed elevated values of alfa amylase 996 U/L, pancreatic amylase 896 U/L, lipase 1820 U/L, glucose 27 mmol/L, alanine transaminase aspartate 575 U/L, aminotransferase 648 U/L, alkaline phosphatase 222 U/L, gamma glutamyl transferase 443 U/L, lactate dehydrogenase 1521 U/L, and CRP 75 mg/L. The abdominal US finding showed only a small amount of intraperitoneal free fluid and increased echogenicity of the omental fat. The pancreatic region could not be visualized by ultrasound owing to overlying gas. The patient was referred for an abdominal CT scan, which showed that most of the pancreatic parenchyma and surrounding fat tissue had been replaced by gas. Pancreatic exudate was present in the omental bursa, left anterior pararenal space, and pelvis with surrounding retroperitoneal fat stranding. Only the distal part of the pancreatic tail remained enhanced on the CT scan, with inflammatory changes in the tail of the pancreas and peripancreatic fat (Figure 1). There were no signs of enteropancreatic fistula. A modified version of the CT severity index was used to assess the severity of acute pancreatitis. After initial evaluation, both the clinical and radiology findings were consistent with emphysematous pancreatitis, i.e., severe form of acute pancreatitis, and the patient was admitted to the intensive care unit for further treatment. He was put on nil per os diet, and a nasogastric tube was placed. In the intensive care unit, the patient was under constant observation from an abdominal surgeon, anesthesiologist, cardiologist, and nephrologist. The progressive worsening of his respiratory function required intubation and mechanical ventilation. His therapy consisted of antibiotics in addition to intravenous fluids, pain control, and nutritional support. Since there was no clinical response to conservative management after several days of clinical worsening and progression of disease, the patient was operated on. Surgical debridement and drainage of the abdominal cavity and omental bursa were performed, as well as feeding enterostomy. Laparotomy revealed diffuse steatonecrosis, destructed pancreas, and abscess collection in the lesser sac, without signs of fistula formation. Multidrug-resistant *Klebsiella pneumoniae* was isolated from the abscess collection in the lesser sac with resistance to 23 tested antibiotics and only intermediate sensitivity to meropenem and sensitivity to tigecycline, chloramphenicol, and colistin. The patient was treated with meropenem, colistin, and tigecycline. In the postoperative period, the patient was hemodynamically unstable and on positive inotropes stimulation with epinephrine and norepinephrine. Unfortunately, after a few days of postoperative treatment in the intensive care unit, the patient was hemodynamically unstable, developed multiorgan failure, and, despite all efforts, died two weeks after his hospitalization.

## 3. Discussion

Necrotizing pancreatitis is characterized by the finding of nonenhancing pancreatic tissue on the CT scan, indicating pancreatic necrosis. It carries a high rate of morbidity and mortality [4]. In the majority of cases, the necrotic tissue remains sterile, but in about 10% to 40% of cases, the necrotic tissue becomes infected [4]. The infection can be monomicrobial or polymicrobial, and organisms reach the pancreatic parenchyma via the bloodstream, lymphatic channels, translocation from adjacent colon, or reflux through the ampulla of Vater [5].

Emphysematous pancreatitis is a rare, potentially fatal subtype of severe acute necrotizing pancreatitis [6]. It represents the presence of gas within or around the pancreas on the ground of necrotizing pancreatitis due to superinfection with gas-forming bacteria [7]. It is usually seen in debilitated patients with uncontrolled diabetes or poor immune function and has poor prognosis, with mortality rates up to 34.5% [1,8]. Most cases have been attributed to infection with Gram-negative organisms, the most common being *Escerichia coli*, *Klebsiella* species, *Pseudomonas*, and *Enterobacter*, with *Clostridium perfringens* being the most common Gram-positive pathogen [9,10].

This entity is diagnosed on clinical grounds and on the basis of radiologic findings [11]. Radiography and ultrasonography have limited sensitivity and specificity in detecting gas in the pancreatic bed [8]. Currently, it is best diagnosed using CT due to its high sensitivity and specificity in the detection of abnormal gas and ability to reliably delineate anatomic location and the extent of the gas [5]. CT scans can show pancreatic necrosis as a nonenhancing pancreatic tissue, gas in the pancreatic parenchyma and peripancreatic space, commonly in the lesser sac, and the involvement of surrounding structures by inflammation [6]. Differential diagnosis includes other causes of air in the pancreas: enteropancreatic fistula, a recent history of endoscopic instrumentation, duodenal diverticulum, penetrating duodenal ulcer, and patulous ampulla of Vater, but these conditions lack features of predominant pancreatic inflammation [8]. In some cases, image-guided fine-needle aspiration from the area of pancreatic and peripancreatic necrosis may be used for microbiological studies and to establish a diagnosis [12]. Diagnosis of infected pancreatic necrosis can be demanding in the absence of aspirate for microbiological analysis, and in some centers, diagnosis is made combining clinical findings, positive blood cultures in the absence of another site of infection, and imaging characteristics, including the presence of gas inclusions in the pancreatic bed [4].

Due to this being a rare entity, guidelines for the treatment of EP are limited and only available from the reported cases and case series. The long-established practice in the treatment of infected pancreatic necrosis was open necrosectomy, with the aim of completely removing the infected necrotic tissue [13]. However, in recent years, management changed towards more conservative measures, consisting of advanced supportive care and interventions like percutaneous drainage and endoscopic therapy [7]. A step-up approach described in the guidelines and other sources in the literature is typically applicable and ranges from conservative management to percutaneous or endoscopic drainage and surgical necrosectomy [14,15].

Conservative management consists of antibiotics in addition to intravenous fluids, pain control, and nutritional support [7]. There are publications of the successful conservative management of EP without any intervention [4]. Evidence for prophylactic antibiotics use in severe pancreatitis is equivocal, but the use of antibiotics is recommended in selected patients with the translocation of intestinal bacteria due to the prolonged duration of hypoperfusion [16]. In our case, CT exam did not show signs of the fistulization of gas-filled collection with the surrounding bowel, as was confirmed after laparotomy. As the pancreatic bed was filled with a large amount of gas, in the absence of a fistula, it was suspected that the cause was superinfection with gas-forming bacteria in this patient with uncontrolled type I diabetes, so prophylactic antibiotics were administered to try to delay intervention and decrease morbidity and mortality.

The image-guided percutaneous catheter drainage of fluid collections as a minimal invasive procedure could be determined on a case-by-case basis and should be added to conservative management or as a temporizing measure prior to surgery. Drainage should be postponed preferably for at least four weeks to allow for the liquefaction of the contents and the development of a fibrous wall, which allows for the removal of content more easily [17]. Several studies have shown that percutaneous drainage was associated with decreased mortality compared to initial surgical management [18,19].

However, nowadays, endoscopic transluminal drainage, which can be followed by endoscopic transluminal necrosectomy (endoscopic step-up approach), is the preferred treatment for symptomatic or infected walled-of necrosis. The aim of the procedure is to gain access to the collection and perform transmural drainage of the content into the gastric/duodenal lumen with plastic or metal stents, after which optionally necrosectomy may be performed through the fistula or stent itself [20]. Chantarojanasiri et al. proposed that endoscopic drainage and necrosectomy in walled-off pancreatic necrosis should be performed in a step-up manner and that the use of novel lumen-apposing stents might be an encouraging treatment option that could reduce the number of steps, procedure time, and the overall number of necrosectomies [21]. The Dutch Pancreatitis Study Group in a multicenter randomized trial concluded that in patients with infected necrotizing pancreatitis, the rate of pancreatic fistulas and length of hospital stay were lower in the endoscopy group, although the endoscopic step-up approach was not superior to the surgical step-up approach in reducing major complications or death [22]. Pattarapuntakul et al. suggested that clinical success rates might be higher, necrosectomy time may be significantly briefer, and the length of hospital stay could be shorter with the use of endoscopic drainage alone compared to combined endoscopic and adjunctive imaging-guided percutaneous drainage [23]. In our patient, based on the CT examination, the pancreatic bed was filled with a large amount of gas, without a significant amount of exudate, which would have been amenable to evacuation with percutaneous drain. Since there was no clinical response to the support measures and acute necrotic fluid collection was not walled off to perform percutaneous drainage, after multidisciplinary discussion, it was decided that the patient should be operated on. Decision was made that percutaneous drainage and the endoscopic approach would not contribute to a satisfactory therapeutic response.

Currently, surgical management also consists of the step-up approach (percutaneous drainage followed, if necessary, by a video-assisted retroperitoneal debridement, and subsequent lavage of the cavity through percutaneous drainage) and is superior to open necrosectomy as a traditional surgical management [22]. This is recommended when the endoscopic approach is not feasible or unsuccessful.

## 4. Conclusions

Emphysematous pancreatitis represents a rare but life-threatening necrotizing infection of the pancreas. It is associated with gas-forming bacteria and is characterized by gas in the pancreatic parenchyma and peripancreatic space. Computed tomography is the preferred imaging modality used to detect this condition. Given its associated high mortality rate, early and prompt recognition and treatment of EP are crucial and require individualized treatment with the involvement of a multidisciplinary team.

## Data Availability

Data are contained within the article.
